# HbA1c variability and the risk of microalbuminuria in patients with type 1 diabetes

**DOI:** 10.1186/1687-9856-2015-S1-P9

**Published:** 2015-04-28

**Authors:** Kyung Chul Song, Ah Reum Kwon, Jung Min Ahn, Hyun Wook Chae, Ho-Seong Kim, Duk Hee Kim

**Affiliations:** 1 Department of Pediatrics, Yonsei University College of Medicine, Republic of Korea; 2 Sowha Children`s Hospital, Republic of Korea

**Keywords:** HbA1c variability, microalbuminuria, type 1 diabetes

## Aims

The higher HbA1c is a risk factor for microvascular complication in type 1 diabetes. However, it remains controversial that glycemic variability seemed to be an additional risk factor for development of complication in diabetes. In this study we have analyzed the HbA1c variability to investigate the influence on progression of microalbuminuria in patients with type 1 diabetes.

## Methods

Fifty patients (M=27, F=23) with type 1 diabetes and microalbuminuria who visited Yonsei University Severance Children’s Hospital were enrolled. In addition, ninety eight (M=31, F=67) type 1 diabetic patients without complication were enrolled for control. Microalbuminuria is defined that urinary albumin excretion rate is between 30 mg/24h and 300 mg/24h. HbA1c during 3 years (just before the development of microalbuminuria or in the past 3 years in control) were reviewed retrospectively. HbA1c variability expressed as standard deviations (SDs) of HbA1c for 3 years.

## Results

There was no difference of mean age between type 1 diabetic patients with microalbuminuria and control (respectively, 22.9±5.5year and 21.6±4.8years, p=0.129). The mean duration to developed microalbuminuria was 9.9±5.1 years. Mean HbA1c was higher in patient with microalbuminuria (14.3±5.1%) than in control (12.2±5.3%, p=0.02). HbA1c variability was also higher in patient with microalbuminuria (1.14±0.81) than in control (0.69±0.38, p<0.001). HbA1c variability was closely related to the mean HbA1c level in all patients (r=0.480, p<0.001). There were also significant trends that microalbuminuria was developed in patients with higher HbA1c SDs in shorter period (r=-0.418, p=0.003).

## Conclusion

This study has shown that HbA1c variability was positively correlated with mean HbA1c level and progression of microalbuminuria. In addition, higher HbA1c variability may shorten the period of development of microalbuminuria. Thus, long-term fluctuation in glycemic control seems to contribute to the development of microalbuminuria in type 1 diabetes.

